# Predictors of pulmonary failure following severe trauma: a trauma registry-based analysis

**DOI:** 10.1186/1757-7241-21-34

**Published:** 2013-04-22

**Authors:** Emanuel V Geiger, Thomas Lustenberger, Sebastian Wutzler, Rolf Lefering, Mark Lehnert, Felix Walcher, Helmut L Laurer, Ingo Marzi

**Affiliations:** 1 Department of Trauma, Hand and Reconstructive Surgery, University Hospital Frankfurt, Goethe-University Frankfurt/Main, Theodor-Stern-Kai 7, Frankfurt am Main, D-60590, Germany; 2 Institute for Research in Operative Medicine, IFOM, University of Witten/Herdecke, Ostmerheimer Str. 200, Cologne, D-51109, Germany

**Keywords:** Multiple trauma, Thoracic trauma, Liver injury, Pulmonary failure

## Abstract

**Background:**

The incidence of pulmonary failure in trauma patients is considered to be influenced by several factors such as liver injury. We intended to assess the association of various potential predictors of pulmonary failure following thoracic trauma and liver injury.

**Methods:**

Records of 12,585 trauma patients documented in the TraumaRegister DGU^®^ of the German Trauma Society were analyzed regarding the potential impact of concomitant liver injury on the incidence of pulmonary failure using uni- and multivariate analyses. Pulmonary failure was defined as pulmonary failure of ≥ 3 SOFA-score points for at least two days. Patients were subdivided according to their injury pattern into four groups: group 1: AIS thorax < 3; AIS liver < 3; group 2: AIS thorax ≥ 3; AIS liver < 3; group 3: AIS thorax < 3; AIS liver ≥ 3 and group 4: AIS thorax ≥ 3; AIS liver ≥ 3.

**Results:**

Overall, 2643 (21%) developed pulmonary failure, 12% (n= 642) in group 1, 26% (n= 697) in group 2, 16% (n= 30) in group 3, and 36% (n= 188) in group 4. Factors independently associated with pulmonary failure included relevant lung injury, pre-existing medical conditions (PMC), sex, transfusion of more than 10 units of packed red blood cells (PRBC), Glasgow Coma Scale (GCS) ≤ 8, and the ISS. However, liver injury was not associated with an increased risk of pulmonary failure following severe trauma in our setting.

**Conclusions:**

Specific factors, but not liver injury, were associated with an increased risk of pulmonary failure following trauma. Trauma surgeons should be aware of these factors for optimized intensive care treatment.

## Background

Several factors such as age [1], base excess [2], number of units of fresh frozen plasma (FFP) transfused [3] and Injury Severity Score (ISS) [4] have been identified as predictors for pulmonary failure in trauma patients. The transfusion of packed red blood cells (PRBC) significantly predicted the development of respiratory complications including pneumonia and acute respiratory distress syndrome (ARDS) [5]. In addition, a lung contusion is known to represent an independent risk factor for acute lung injury (ALI), ARDS and pulmonary failure [6,7], with the incidence of pulmonary failure increasing if the volume of pulmonary contusion exceeds 20 per cent of the total lung volume [8]. However, pulmonary failure is a major contributor to morbidity and mortality in trauma patients.

Abdominal trauma and solid organ injuries can result in a significant need for PRBC transfusion leading secondarily to pulmonary failure. In particular, when non-operative management in blunt hepatic injuries fails, several complications such as pneumonia, bacteremia and ARDS can occur [9]. Nevertheless, the impact of liver injury on the incidence of pulmonary failure in multiple trauma patients remains unclear.

Thus the goal of the present study was to test the hypothesis that patients sustaining significant thoracic trauma in combination with a relevant liver injury are more likely to develop pulmonary failure when compared to patients sustaining thoracic trauma without concomitant liver injury. In addition, we intended to analyze risk factors for the development of pulmonary failure in severely injured trauma patients.

## Methods

### TraumaRegister DGU^®^ of the German Trauma Society (TR-DGU)

The TraumaRegister DGU^®^ of the German Trauma Society is a multi-center database, where severely injured patients are prospectively documented at standardized time points: (1) pre-hospital phase: mechanism of injury, initial physiology, first therapy, neurological signs, prehospital time; (2) emergency room (ER): physiology, laboratory findings, suspected pattern of injury, therapy, time sequence of diagnostics; (3) intensive care unit (ICU): status on admission, organ failure, sepsis, duration of ventilation; and (4) final outcome: hospital stay, survival, complete list of injuries including anatomic injury assessment using the ISS [10], operative procedures, and pre-existing medical conditions (PMCs). Interventions are documented according to the International Classification of Procedures in Medicine (current documentation sheets and participating centers available on http://www.traumaregister.de). Data collection started in 1993 by the Working Group on Polytrauma of the German Trauma Society to evaluate the quality of trauma care in Germany, Austria, the Netherlands and Switzerland [11]. All variables are continuously entered into a web based data server. Patients eligible are those suspected to require ICU treatment after trauma and to present an ISS ≥ 16 or those that die in the emergency room. Data are submitted to a central database hosted by the Institute for Research in Operative Medicine (IFOM) at the University of Witten/Herdecke in Cologne, Germany. Data anonymity is provided both for the individual patient as well as the participating hospital [12-14]. The TR-DGU is approved by the review board of the German Trauma Society and is in compliance with the institutional requirements of its members. The TR-DGU comprises datasets of 29,353 patients documented between 1993 and 2006 from 125 participating hospitals.

### Patients

Patients documented between 1993 and 2006 in the TR-DGU were analyzed for eligibility. Inclusion criteria were: (1) ISS ≥ 16, (2) primary admission, (3) survival ≥ 24 hours, and (4) information available regarding (a) pulmonary failure, (b) administration of PRBC and (c) duration of mechanical ventilation. An injury was graded as severe if an Abbreviated Injury Scale (AIS) ≥ 3 was present [15]. According to the injury pattern, patients were subdivided into the following subgroups: group 1 included multiple trauma patients, who had sustained neither a thoracic trauma nor a severe liver injury (AIS thorax < 3; AIS liver < 3); group 2 consisted of patients who had sustained a multiple injury including thoracic trauma without any liver injuries (AIS thorax ≥ 3; AIS liver < 3); multiple trauma patients who had sustained a relevant liver injury without concomitant thoracic trauma (AIS thorax < 3; AIS liver ≥ 3) were assigned to group 3 and ultimately patients with significant thoracic trauma and liver injury were assigned to group 4 (AIS thorax ≥ 3; AIS liver ≥ 3).

### Selection of variables

The selection of potential predictors for the development of pulmonary failure was based on previous reports and included: age [16], sex [17], ISS [18] and New Injury Severity Score (NISS) [19], maximum AIS scores for thorax, abdomen, extremities and head, pre-hospital infusion volume [20], number of PRBCs transfused [18] and infusion volume in the ER until ICU admission [21], rate of multi organ failure (MOF) [22], defined as organ failure of two systems of ≥ 3 SOFA-score points of ≥ 2 days duration [23], duration of mechanical ventilation [20], ICU and hospital length of stay.

The following factors were dichotomized for univariate and multivariate logistic regression analysis: AIS thorax ≥ 3 versus < 3, administration of PRBCs versus non-administration, PRBC ≥ 10 versus < 10, relevant thorax injury versus no thorax injury, relevant liver injury versus no liver injury, AIS head ≥ 3 versus ≤ 1, AIS abdomen ≥ 3 versus ≤ 1, presence versus absence of PMCs, and male versus female gender.

### Outcome evaluation

The primary outcome parameter was the incidence of pulmonary failure defined by pulmonary failure for at least two days according to Vincent et al. [23]. Pulmonary failure was assumed if the Sepsis-related Organ Failure Assessment (SOFA) score was ≥ 3 points for a minimum duration of two days.

### Statistical analysis

The demographic and clinical characteristics comparing the previously described groups were evaluated using bivariate analysis. The *p* values for categorical variables were derived from the Chi-square or 2-sided Fisher’s exact test and for continuous variables from the Student's or the Mann-Whitney test. Multivariate analysis was performed to control for confounders diverging significantly (*p* < 0.05) between the compared groups. For continuous outcomes, analysis of covariance was used to adjust for confounders that were significant at *p* < 0.05.

To identify risk factors that were independently associated with the presence of pulmonary failure, a stepwise logistic regression model was utilized and risk factors from the bivariate analysis with a *p* value < 0.2 were included in the model.

Variables are given as mean ± standard deviation (SD) and as number and percentage for categorical variables. Odds ratios with 95% confidence intervals (CI_95_) were calculated for statistically significant variables. Statistical analysis was performed using the Statistical Package for Social Sciences (SPSS Windows^©^), version 15.0 (SPSS Inc., Chicago, IL, USA).

## Results

Out of the 29,353 patients documented in the Trauma Registry, 12,585 patients (43%) with a mean age of 40.8 ± 19.7 years and a mean ISS of 28.6 ± 11.1 points fulfilled the inclusion criteria and were enrolled in this study. The basic characteristics of the study groups are summarized in Table 1. The overall rate of pulmonary failure was 21% (n = 2,643). The largest proportion of patients who developed pulmonary failure was found in group 4 (36%; n = 188). Since ISS and other variables differed significantly between the groups, a direct comparison between the groups regarding the primary endpoint “pulmonary failure” is difficult to interpret (Table 1).

**Table 1:** Basic characteristics: patients were assigned to four different groups according to their AIS lung and AIS liver

	**Group 1**	**Group 2**	**Group 3**	**Group 4**
	**AIS lung < 3**	**AIS lung ≥ 3**	**AIS lung < 3**	**AIS lung ≥ 3**
	**AIS liver < 3**	**AIS liver < 3**	**AIS liver ≥ 3**	**AIS liver ≥ 3**
	**(n = 5347; 42.5%)**	**(n = 6528; 51.9%)**	**(n = 188; 1.5%)**	**(n = 522; 4.1%)**
Age, years (mean ± SD)	42.1 ± 21.1^a^	40.5 ± 18.6^c^	31.1 ± 14.2^d^	34.8 ± 16.8^e^
ISS	24.1 ± 9.1	31.3 ± 11.1	27.4 ± 9.5	40.8 ± 11.6
NISS	31.5 ± 12.9	36.1 ± 12.8	34.6 ± 12.3	44.8 ± 12.6
Duration of ventilation (days)	7.3 ± 12.1	10.1 ±12.8	8.3 ± 11.2	13.7 ± 17.4
ICU length of stay (days)	11.5 ± 14.6	15.1 ± 15.8	13.1 ± 13.2	19.8 ± 22.3
In hospital length of stay (days)	28.5 ± 31.7^b^	32.2 ± 35.3	29 ± 24.5^d^	35 ± 29.9^f^
MOF (%) (defined as organ failure of two systems of >2 SOFA-score points of ≥ 2 days duration)16	19 ± 39	25 ± 43	26 ± 44	39 ± 49
OF (%)	35	44	38	55
In hospital mortality rate (%)	13	9	9	14
Pulmonary failure ≥ 2 days (%)	12	26	16	36

Abbreviations: ISS, injury severity score; AIS, abbreviated injury scale; ICU, intensive care unit; MOF, multi-organ failure; OF, organ failure; SD, standard deviation; missing values: ^a^0.4%, ^b^1.5%, ^c^0.3%, ^d^0.5%, ^e^0.8% and ^f^1.7%.

Comparing patients who developed pulmonary failure and patients who did not, both cohorts differed significantly with respect to age, ISS, NISS, duration of mechanical ventilation, ICU length of stay, length of stay in hospital, the number of PRBCs transfused, pre-hospital volume substitution and volume substitution in the ER (Table 2). Hence, we performed a univariate analysis to evaluate the potential impact of different factors on the incidence of pulmonary failure (Table 3).

**Table 2:** Bivariate analysis of selected parameters in patients with and without pulmonary failure

	**Total**	**No pulmonary failure**	**Pulmonary failure**	**P-value**
	**(n = 12,585 )**	**(n = 10,000)**	**(n = 2,585)**	
Age	40.8 ± 19.7	40.1 ± 19.6	43.7 ± 19.7	<0.0001
ISS	28.6 ± 11.1	27.3 ± 10.5	33.7 ± 12.1	<0.0001
NISS	34.5 ± 13.2	33 ± 12.7	40.1 ± 13.5	<0.0001
Length of ventilation (days)	9.0 ± 12.8	6.4 ± 10	19.4 ± 16.5	<0.0001
Stay in ICU (days)	13.7 ± 15.8	10.9 ± 13.2	24.91 ± 19.5	<0.0001
In hospital stay (days)	30.7 ± 33.5	28.1 ± 32.5	40.6 ± 35.4	<0.0001
PRBC before ICU adminission (Units)	2.5 ± 6.2	2.0 ± 4.9	4.8 ± 9.4	<0.0001
Prehospital volume substitution (ml)	1,414 ± 1,155	1,338 ± 1,106	1,707 ± 1,285	<0.0001
Volume ER	2,948 ± 3,061	2,702 ± 2,645	2,947 ± 3,061	<0.0001

Abbreviations: *ISS*, Injury Severity Score; *ICU*, Intensive Care Unit; *PRBC*, Packed Red Blood Cells; *ER*, Emergency Room.

**Table 3:** Incidence of pulmonary failure in univariate conditions

	**Pulmonary failure**		**P-value**
**Parameters**	**No [%]**	**Yes [%]**	
PMCs	18.7	26	<0.0001
ISS ≥ 25	11.5	26.6	<0.0001
Sepsis	15.5	58.1	<0.0001
Early termination of regular ER diagnostics due to emergency operation	19.4	32	<0.0001
Glasgow coma scale ≤ 8	18	26.7	<0.0001
Lung injury (AIS_thorax_ ≥3)	12.6	26.8	<0.0001
Liver injury (AIS_adomen_ ≥3)	19.9	31.1	<0.0001
Male gender	16.2	22	<0.0001
Blunt trauma	17	20.7	0.051
Age ≥ 60 years	19.4	25	<0.0001
AIS_head_ ≥ 3	20.2	20.8	0.387
AIS_thorax_ ≥ 3	12.6	25.9	<0.0001
AIS_abdomen_ ≥ 3	18.9	26.1	<0.0001
AIS_extremity_ ≥ 3	18.7	23.4	<0.0001
Isolated TBI	21.9	10.6	<0.0001
Prehospital blood pressure ≤ 90 mmHg	18.9	28.4	<0.0001
Blood pressure in the ER ≤ 90 mmHg on admission	19.2	32.5	<0.0001
Administration of PRBC	16	30.3	<0.0001
Ttransfusion of ≥ 10 PRBC s	18.8	39.1	<0.0001
Surgical interventions	10.8	22.4	<0.0001
Admission during night shift	20.9	19.5	0.106

Abbreviations: *PMC*, Pre-existing Medical Condition; *ISS*, Injury Severity Score; *ER*, Emergency Room; *AIS*, Abbreviated Injury Scale; *TBI*, Traumatic Brain Injury; *PRBC*, Packed Red Blood Cell.

After excluding cases with missing variables, multivariate forward logistic regression analysis was subsequently performed in a subset of 9,920 patients. Those factors which proved to show a significant correlation with the incidence of pulmonary failure were included in the multivariate analysis. The presence of relevant lung injury, male gender, PMCs, transfusion of more than 10 PRBCs as well as ISS and age were identified as predisposing factors that were independently associated with the development of pulmonary failure (Table 4). In contrast to our hypothesis, however, liver injury did not prove to be an independent predictor of pulmonary failure.

**Table 4:** List of independent predictors for pulmonary failure as dependent variable in multivariate logistic regression analysis

**Risk factor**	**p-value**	**Odds ratio (CI**_**95**_**)**
Lung injury (AIS_thorax_ ≥3)	<0.0001	1.961 (1.745-2.205)
Male gender	<0.0001	1.654 (1.460-1.874)
PMCs	<0.0001	1.581 (1.401-1.784)
Transfusion of ≥10 PRBC	<0.0001	1.418 (1.190-1.690)
Administration of PRBC	<0.0001	1.386 (1.228-1.564)
Glasgow coma scale ≤ 8	<0.0001	1.273 (1.126-1.439)
ISS per point	<0.0001	1.027 (1.022-1.032)
Age per year	<0.0001	1.015 (1.012-1.017)

Abbreviations: *PMC*, pre-existing medical condition; *PRBC*, Packed Red Blood Cells; *ISS*, Injury Severity Score.

## Discussion

In this retrospective study evaluating 12,585 multiple traumatized patients, the presence of concomitant liver injury in thoracic trauma had no impact on the development of pulmonary failure. However, we found several factors which revealed a significant association with the incidence of pulmonary failure confirming previously published findings [8,24]. In the current analysis, the presence of lung injury (AIS thorax ≥ 3) and other PMCs, sex, and the administration of more than 10 PRBCs increased the incidence of pulmonary failure following thoracic trauma.

To the best of our knowledge, this is the first study addressing the question of whether relevant liver injury in patients with thoracic trauma has a significant impact on clinical outcome in terms of pulmonary failure.

Pulmonary failure is a well-known complication after multiple trauma and has also been described following major hepatic surgery with an incidence of up to 82% [8,24]. It is commonly associated with poor survival and quality of life, a significant increase in morbidity as well as increased health care costs [25]. There is growing evidence that organ interactions must be taken into account to understand the determinants of ARDS. Among extra-pulmonary organs, the liver plays a central role in regulating cytokine kinetics relevant to acute lung injury. Since various studies in both, ARDS patients as well as ALI rodent models, revealed elevated leukotriene (LT) levels in lung edema fluid, it is proposed that LT levels have a substantial impact on the pathophysiology of ARDS [26-29]. The liver is the principle organ for metabolic leukotrien B4 [LTB4] inactivation. Impairment of liver function, e.g. due to relevant abdominal trauma, may therefore result in a limited and/or delayed catabolism of LTB4, which ultimately increases the risk of ARDS as well as pulmonary failure.

In addition, it has been shown that there is a high incidence of sepsis-induced acute lung injury in patients with end-stage liver failure emphasizing a close correlation between ARDS and liver failure [30]. Hence, one can expect that additional liver injury following thoracic trauma promotes the incidence of pulmonary failure and ARDS, respectively. Unlike Matuschak and co-workers in their cohort of 29 patients with end-stage liver failure, we did not find liver injury to be a significant trigger for pulmonary failure in thoracic trauma when applied to the data of the TR-DGU in our setting.

As reported two decades ago in a series of 185 patients with blunt traumatic hepatic injury, class I-III hepatic injuries are relatively negligible as a cause of death or serious complications. In these populations the associated patterns of organ injury, such as brain and chest injuries are more likely to play a major role in the induction of serious complications, i.e. ARDS, which is frequently associated with high mortality [31]. Although in our population liver injury was simply classified according to the AIS score and information regarding the classification of Moore [32] was missing, we likewise found severe liver injury, defined as AIS liver ≥ 3, not to be significantly associated with the development of severe pulmonary complications following thoracic trauma. Comparing thoracic trauma patients with and without liver injury did not reveal a statistically significant difference in the incidence of pulmonary failure and the overall in-hospital mortality.

Patients with relevant lung injuries had a higher risk of developing pulmonary failure (odds ratio 2.03, CI_95_ 1.81-2.29), which is in accordance with the literature [33,34].

Massive transfusion is commonly suggested as a major risk factor for the development of ARDS and pulmonary failure due to interaction between non-specific systemic inflammatory mediators, anti-granulocyte antibodies or depressed immune response [35-37]. We were able to confirm that the transfusion of ≥10 units of PRBCs until ICU admission is an independent predictor for the development of ARDS, as previously reported by Chaiwat and co-workers [38]. They further demonstrated that one additional unit of PRBCs in patients receiving more than 10 units accounted for a 6% higher risk of ARDS [38]. However, in our analysis, liver injury (AIS ≥ 3) was not associated with pulmonary failure but revealed a significant association with transfusion of PRBCs, whereas transfusion of PRBCs was significantly associated with pulmonary failure.

### Limitations

Although this is one of the largest studies investigating the association of liver injury and the development of pulmonary failure, there are some methodological issues and limitations, the most important being the retrospective nature of data analysis. All data were obtained from the database of the German Trauma Society, which was founded in the early 1990s. This database is not specifically developed for the documentation of patients with thoracic and/or liver trauma, but rather for the documentation of multiply traumatized patients in general. Moreover, we excluded patients who experienced pulmonary failure for only one day, because many trauma patients require initial lung function support during the first hours in hospital and on ICU. However, data were recruited from more than 120 hospitals in Germany, Austria, Switzerland and the Netherlands. Since we included almost 13,000 patients we believe that the patient population is representative.

Finally, the applied definitions of PMCs, for example, may vary widely among the different participating hospitals. While some diseases were clearly defined in our setting, others should be more specified in future studies. Although specifically designed clinical studies could reveal much more detail of the disease and its consequences, registry studies are from our point of view still considerable, since a large and representative number of patients is available for the analysis of even minor effects.

## Conclusions

To conclude, in the current analysis, liver injury did not prove to be independently associated with the presence of pulmonary failure. Basic trauma research should focus on those identified risk factors to further explore their impact and pathophysiology to approach an improved and individualized therapeutic regime.

## Competing interests

The authors declare that they have no competing interests. The TraumaRegister DGU^®^ of the German Trauma Society (Deutsche Gesellschaft für Unfallchirurgie, DGU) was partly funded by the Deutsche Forschungsgemeinschaft (DFG) Ne 24 385/5 and by a grant of Novo Nordisk A/S, Bagsvaerd, Denmark.

## Authors’ contributions

EG, TL, SW and RL conceived and designed the study. FW, ML, HLL and IM supervised the conduct of the trial. SW, EG and RL collected data. RL, SW, EG and FW provided statistical advice on study design and analyzed the data; FW, ML, HLL and IM participated in design and coordination of the study. EG, TL and SW drafted the manuscript. All authors read and approved the final manuscript.
